# The MULTI-ACT model: the path forward for participatory and anticipatory governance in health research and care

**DOI:** 10.1186/s12961-022-00825-2

**Published:** 2022-02-17

**Authors:** Paola Zaratin, Deborah Bertorello, Roberta Guglielmino, Danilo Devigili, Giampaolo Brichetto, Valentina Tageo, Gabriele Dati, Stephanie Kramer, Mario Alberto Battaglia, Monica Di Luca

**Affiliations:** 1 Italian Multiple Sclerosis Society Foundation, Genova, Italy; 2 Collectibus Srl Società Benefit, Milano, Italy; 3 Wise Angle Consulting SL, Barcelona, Spain; 4 European Brain Council, Brussels, Belgium; 5 Laboratory of Pharmacology of Neurodegeneration-DiSFeB at the University of Milano, Milano, Italy; 6 Department of Life Sciences, University of Siena, Siena, Italy

**Keywords:** Participatory governance, Mission-oriented research, Patient engagement, Co-accountability, Responsible research and innovation

## Abstract

The COVID-19 pandemic has unmasked even more clearly the need for research and care to form a unique and interdependent ecosystem, a concept which has emerged in recent years. In fact, to address urgent and unexpected missions such as “fighting all together the COVID-19 pandemic”, the importance of multi-stakeholder collaboration, mission-oriented governance and flexibility has been demonstrated with great efficacy. This calls for a policy integration strategy and implementation of responsible research and innovation principles in health, promoting an effective cooperation between science and society towards a shared mission. This article describes the MULTI-ACT framework and discusses how its innovative approach, encompassing governance criteria, patient engagement and multidisciplinary impact assessment, represents a holistic management model for structuring responsible research and innovation participatory governance in brain conditions research.

## Introduction

The profound sign that the COVID-19 crisis should ultimately make clear is the recognition and more relevant role of organizations that apply responsible research and innovation (RRI) and therefore operate in the collective interest [1]. Among these, nonprofit organizations have demonstrated strategic skills in dealing with the COVID-19 pandemic, carrying out a subsidiary task with respect to the government, and integrating strategic skills not only in health and social care but also in research [2]. Introduced into the debate about a decade ago [3], RRI aims to align the processes and outcomes of research and innovation (R&I) with societal values by involving the broad range of stakeholders from a very early stage [4, 5]. The European Commission (EC), one of the larger funders of science and societal interrelationship in research development, invested heavily in the inclusion of RRI in its Horizon 2020 Framework Programme, under the heading “Science with and for Society” programme (SwafS) [6]. According to a recent article [7], the European Union (EU) promotes RRI in principle, but implementation leaves much to be desired, and the authors indicate that much effort should be directed towards improving the policy integration strategy and implementation. An important driver of this change lies in the EU recommendations to promote a systematic integration of EU RRI project outcomes towards institutional change and a better social contract [8].

Within this strategic framework, the MULTI-ACT project [9] is one of the projects in the European RRI portfolio funded under the “New constellations of changing institutions and actors” call (European Commission Horizon 2020 Work Programme 2016–2017, Swafs-05-2017). MULTI-ACT aims to increase the impact of health research on citizens with brain conditions, as well as their families and caregivers, through an innovative participatory and anticipatory governance model: a Collective Research Impact Framework (CRIF) allowing for the effective co-accountability of all relevant stakeholders in meeting the transformational missions for brain conditions. According to a European RRI portfolio classification [10] and a recent analysis [11], MULTI-ACT has the potential to enable institutional changes needed for applying RRI participatory governance in health research.

In this article we describe the MULTI-ACT CRIF and discuss how its unique holistic concept, encompassing governance, patient engagement and multidimensional impact assessment, represents a managerial tool for structuring an RRI participatory and anticipatory governance model for collective sustainability of transformational health missions, such as those in brain conditions.

The inspirational principle of the MULTI-ACT model is to enable institutions to “act like an organization, but think like a movement” [12].

### Design with the end/impact in mind: mission-oriented research and the case of brain conditions

The COVID-19 pandemic has unmasked the need for health research and care to form a unique and interdependent ecosystem [13] to provide the needed resilience, enhancing the adaptability to unexpected changes [14, 15] towards more personalized care. As also indicated in the 2016 consensus document of the Horizon 2020 Scientific Panel for Health [16], healthcare is not only the consequence of research, but also the setting for research. Rising to this challenge lies precisely in our ability to leverage the insights gained from the RRI models and tools [8], which can make the relevant stakeholders co-accountable for a shared mission (mission-oriented research) and a coordinated agenda.

In particular, multi-stakeholder research initiatives are essential to delivering the transformational missions demanded by health research. Within this strategic framework, research institutions must make themselves capable of rethinking their own governance and working models through an enhanced collaborative sustainable approach. The ability of RRI to spur the alignment of the processes and outcomes of R&I with societal values has long been recognized and well documented [8, 10]. Less recognized is the fact that RRI also needs a direction. A previous relevant responsible innovation in health (RIH) framework has provided important insights [17] for defining the dimensions that specifically characterize RIH. However, to further help in framing the direction of RRI in health, innovative models to enable mission-oriented participatory governance [18, 19] are needed. This is expected to promote anticipatory governance as well [20]. Indeed, recent studies recognized an increasing perception of the need to revise the usual mechanisms of governance of science for anticipating and managing risks and opportunities, especially in periods of great crisis.

One of the innovative aspects of the MULTI-ACT framework versus existing models is that it considers the mission-related dimension as one explicit driver for co-accountability of the stakeholders involved. The framework introduces the evaluation of the efficacy of an R&I initiative interpreted as its capacity to fulfil the shared mission (along with the other impact dimensions detailed hereafter) as a pivotal element to promote research that has an impact on patients and society. The circularity and flexibility of the MULTI-ACT framework aims to aid institutions in applying participatory governance to maximize the success of fulfilling the mission and achieving anticipatory governance to manage emerging knowledge-based evidence while such management is still possible [20].

The integration of RRI mission-oriented participatory and anticipatory governance is particularly urgent in the field of brain conditions [21], as also revealed by the COVID-19 pandemic [22].

Brain conditions, mental and neurological alike, account for a large burden on the European population. In 2017, 307.9 million neurological disease diagnoses alone were counted in the 28 EU countries—540.3 million neurological diseases in the WHO European Region. Furthermore, it has been estimated conservatively that every year, 27% (neurological condition prevalence [23]) of the total adult EU population is affected by a mental disorder, amounting to over 82.7 million affected persons. Researchers and physicians have noted growing evidence over the past several months of a major and dramatic impact of long COVID-19 on brain health, with severe mental and neurological consequences [24]. The direct neurological impact of the virus has begun manifesting in more than two thirds of patients with COVID-19 [25], with physicians working to better understand exactly how COVID-19 has affected their patients [26]. Halving the human burden of brain conditions would mean a tremendous impact in terms of improvement in quality of life for patients and their families and caregivers. Currently, brain disorders are estimated to account for global cost (direct and indirect) exceeding €800 billion for national health budgets [27].

A “big-thinking Brain Mission” [28] that involves all relevant stakeholders is required to meet the complex and diverse challenges of brain disorders and to help society cope, and it will also be an economic game changer [29, 30]. Investment in research on neurological conditions will not only help increase life expectancy and reduce suffering but will also result in significant savings for social and care services. Despite encouraging advances in diagnosis and therapy, the complexity of the brain demands a redoubling of the effort to capitalize on scientific results, which requires engagement of the stakeholders in an integrated brain mission envisaged at “a scale similar to the Space Race” [28].

### MULTI-ACT: a new Collective Research Impact Framework

In a time of challenges that call for transformational missions in brain conditions, the future of health research sustainability requires new RRI multi-stakeholder and multidisciplinary managerial models [31–33]. “Monitoring the evolution and benefits of RRI” has also highlighted as an unmet need the existence of formal governance models for RRI within health research organizations [34, 35].

An important driver of this change should be a paradigm shift towards a co-accountability approach [36], engaging multiple stakeholders to define flexible impact assessment systems that enable the consideration and alignment of a plurality of perspectives towards a given mission [37]. Meeting this challenge will require innovative approaches in achieving an impact on the excellence and economic dimensions [38, 39], but first of all on the social and patient-reported dimensions. The thinking behind RRI indeed seeks to challenge our notion of good science as such. It argues that excellence, validity and relevance are connected by engaging patients as key stakeholders in the research continuum [40, 41].

**Fig. 1:**
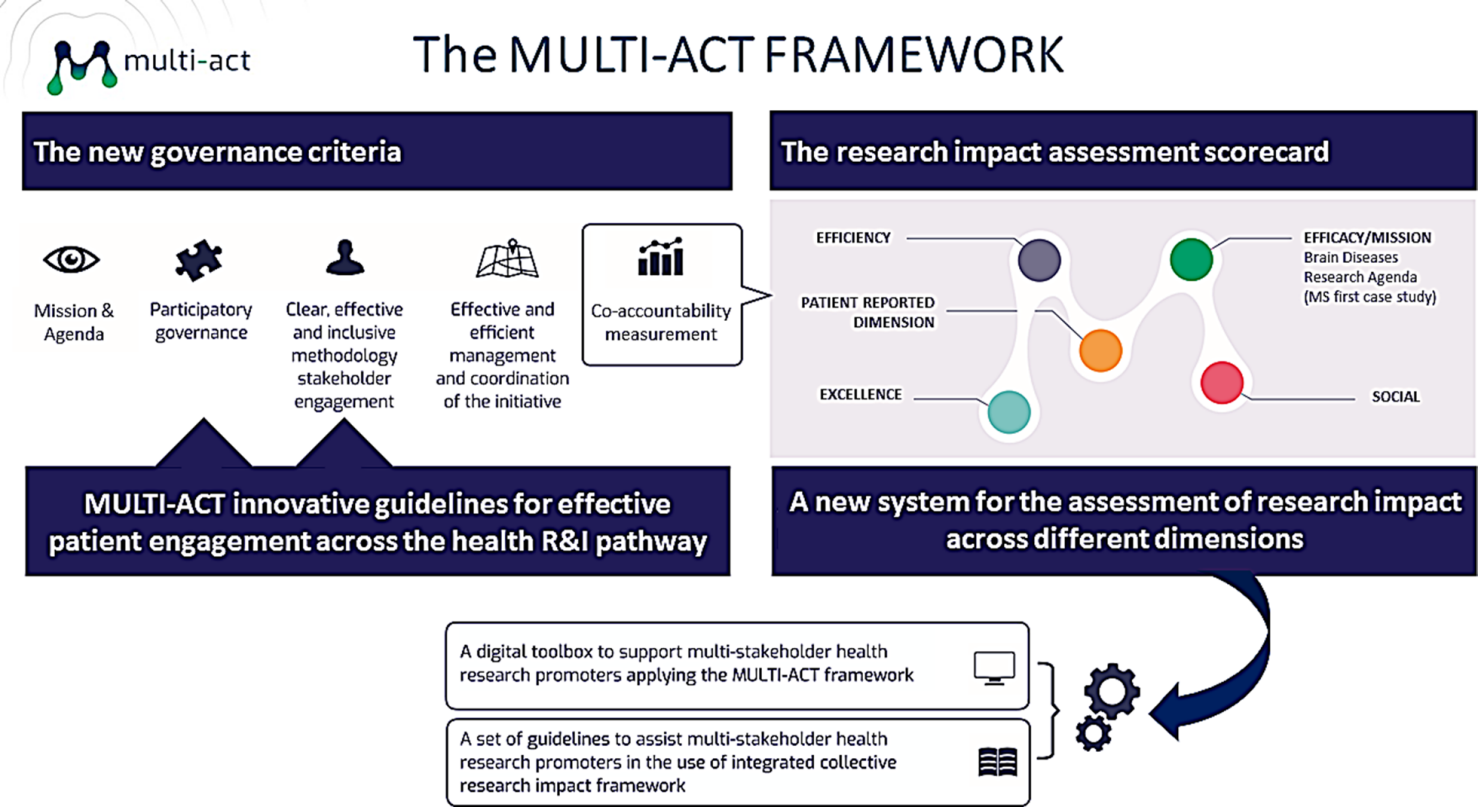
The MULTI-ACT strategic framework

Within this strategic intent, MULTI-ACT CRIF enables an innovative co-accountability strategy which is translated into new governance criteria, including innovative guidelines for effective patient engagement across the health R&I pathway, and a new system for the assessment of research impact across different dimensions (Fig. 1).

**Fig. 2:**
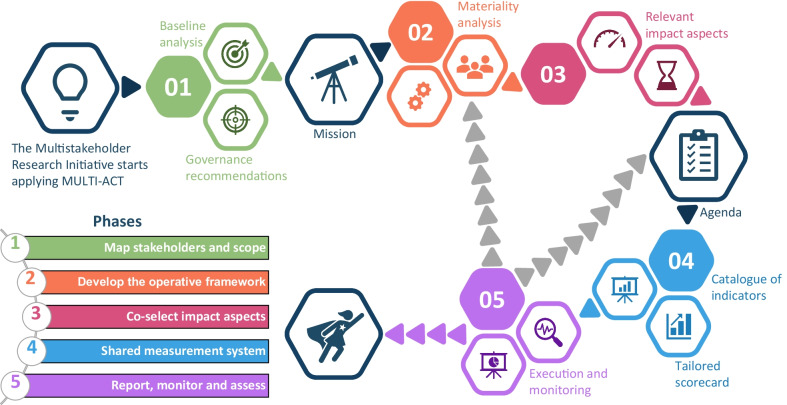
The MULTI-ACT CRIF user journey through the five phases

The MULTI-ACT CRIF is made available to the R&I community through a free and user-friendly digital toolbox (accessible via the link https://toolbox.multiact.eu/). A workflow guides the user in the adoption and implementation of MULTI-ACT CRIF [42] (Fig. 2). In the MULTI-ACT model, engaging patients as key stakeholders [11, 43] (science with patient input) and measuring the impact of research on outcomes that matter to them (science of patient input) becomes instrumental in making stakeholders co-accountable for the mission and the agenda of brain conditions, and then enabling a unique health research and care ecosystem.

The workflow (Fig. 2) that an institution is expected to follow to adopt the MULTI-ACT CRIF comprises five main phases: (1) mapping of stakeholders and establishing the scope and the mission, (2) developing an operative framework, (3) co-selection of relevant impact aspects and agenda definition, (4) shared measurement system (selection of indicators) and (5) reporting, monitoring and assessment. The phases build on five theoretical co-accountability pillars [36].

### The MULTI-ACT CRIF user journey

The health research community has already demonstrated an increasing interest in the model [38, 41, 44]. The MULTI-ACT digital toolbox can guide the funding and performance of health research and care organizations in the application of the MULTI-ACT workflow that is composed of five main phases (displayed in Fig. 2).

Each research initiative must define its scope and mission (phase 1) and implement an operating framework for its realization (phase 2). The control of the results is entrusted to the definition of specific aspects that lead to the definition of an agenda (phase 3) and which are the basis for the selection of the related multidimensional indicators of the measurement model shared by the stakeholders involved in the initiative (phase 4).

The collective materiality analysis is the innovative managerial tool that MULTI-ACT makes available, also through the digital toolbox, in order to provide stakeholders with the ability to jointly identify and co-select the crucial aspects.

By selecting the different aspects, each stakeholder also has the opportunity to express the expected return of engagement and investment on a given shared mission and agenda. In order to constitute the dashboard of the initiative (shared measurement system), the toolbox recommends the use of a manageable number of indicators (at least two from each dimension), ensuring a balance in stakeholder return on investment: each will have a scorecard of 12–15 aspects chosen from a list of 53 aspects available, and 12–15 relevant indicators chosen from the 125 that the model makes available in its impact assessment scorecard [42]. Finally, the continuous monitoring of the indicators provides the basis for corrective and anticipatory actions (phase 5) to be made in order to ensure that the agenda is monitored to meet the mission. For each of the phases described above, MULTI-ACT has defined specific operational tools that make up the content of the three components of the model: governance criteria, patient engagement and impact assessment [43].

The circle closes with the publication of the periodic report of the initiative, which MULTI-ACT suggests should be produced annually, and which provides the basis for the analysis of the differences between what was planned and what was achieved, allowing for the identification of the appropriate improvements in the agenda of the initiative. Indeed, for a mission-oriented approach, while the mission is defined at the beginning of the initiative, the alignment of the agenda to the mission needs to be monitored and checked regularly, and therefore, phases 2 to 5 should be repeated accordingly (e.g. on an annual basis). This reflects the circularity and flexibility of the MULTI-ACT framework needed to enable participatory but also anticipatory governance.

The management approach of the entire process (phases 1 to 5) and the application of its operational tools must be based on the constant involvement of the stakeholders—in particular the patients, their families and caregivers, as key stakeholders in health research—according to the principles and indications provided by the MULTI-ACT patient engagement guidelines [46].

### MULTI-ACT participatory and anticipatory governance: science with and of patient input

The innovative contribution of MULTI-ACT, starting from the analysis of over 100 collective impact initiatives, was to develop a governance model that included the criteria and rules to ensure the best operating conditions for multi-stakeholder initiatives. By carrying out a context and literature analysis of multi-stakeholder initiatives, MULTI-ACT, in line with its vision and objectives in terms of responsible governance, identified a set of five criteria and 19 sub-criteria [45] to support the application of the MULTI-ACT co-accountability phases.

At the core of the new participatory governance criteria of MULTI-ACT are new guidelines to enable patient engagement. The holistic approach of MULTI-ACT is instrumental in achieving an effective patient engagement strategy. In fact, the MULTI-ACT patient engagement strategy is not a stand-alone strategy; it is empowered by the other two components of the model (i.e. governance criteria and multidisciplinary impact assessment), allowing all the stakeholders to co-create with the patients (including their families and caregivers), to acknowledge the value of patient input, and to align their interests with those of the patients, towards a common mission and shared agenda. The value of this holistic engagement relies on recognizing the patients as research team members bringing their “experience of the disease” into the team, complementing existing approaches for educating patients to act as scientists.

Indeed, MULTI-ACT provides guidance and tools for providing the needed skills, knowledge and competence for patients to participate in the research team together with all the other stakeholders, with their specific and valuable “experiential” assets. The three innovative assets of the MULTI-ACT patient engagement guidelines [46] are (i) the Engagement Coordination Team, an innovative governance body to ensure stakeholder representativeness and co-accountability for patient engagement; (ii) a training focused on how to empower patients to cooperate and to integrate their experiential knowledge into the research, complementing existing training to make patients “experts” [47]; and (iii) the importance of understanding and measuring the impact of R&I on outcomes that matter to patients (patient-reported dimension) as core and transverse dimensions of the co-accountability model.

Over the last decade, patient engagement has become more important along with the democratization of health sciences. Patients started to be engaged not only in a passive role, but also as co-researchers. What began as an extension of patient advocacy [48] has now evolved into an emerging scientific discipline aimed at understanding and incorporating patient experiences, needs, expectations and perspectives (patient experiential knowledge) [40, 41, 49, 50] into the process of health research.

MULTI-ACT performed a landscape analysis [51] in order to assess existing experiences of patient engagement in research and found that much of the current guidelines for patient engagement focus on enabling “expert patients” in the “medicines life cycle”. MULTI-ACT proposes a complementary strategy: a roadmap to capture the experiential knowledge of patients that complements the expertise of the other stakeholders and can be acknowledged and used as a valuable asset for research and care [52] (science with patient input).

The big challenge for patient engagement is always to ensure representation of the patient community. A new governance body proposed by MULTI-ACT, the Engagement Coordination Team, ensures the representativeness of the relevant community and is in charge of turning individual patient perspectives into a collective one. Within this frame, patient advocacy organizations are playing an important role, as boundary organizations, to define and implement the “how to” that enables this transition.

In line with the MULTI-ACT guidelines, engaging patients as key stakeholders will enable us to measure the impact of research on outcomes that matter most to patients (science of patient input), making health research and care more sustainable. Patient-reported outcome measures (PROMs) included in the MULTI-ACT Master Scorecard (MSC) [53] are increasingly instrumental in making stakeholders co-accountable for patient engagement in brain conditions research and care. “The use of PROs is especially challenging for brain conditions, considering that patients are usually old, fragile, with comorbidities, and often have cognitive or communication impairments” [44]. However, Parkinson’s, multiple sclerosis, stroke and mild cognitive impairment, which significantly affect brain disorder burden [54], are amenable to such patient-based feedback. Moreover, digital technology has the potential to bring passive measures of the individual’s perception and feelings to the point of research and care, facilitating PROMs collection. Building also on MULTI-ACT best practice, the global Patient Reported Outcomes Initiative for Multiple Sclerosis (PROMS) has been launched to tackle the challenges [55].

The MULTI-ACT project developed a high-level policy-oriented document addressing key actions (see Table 1) to be taken in the short, medium and long term by policy-makers and R&I funders to implement MULTI-ACT patient engagement guidelines [56].

**Table 1:** The MULTI-ACT patient engagement guidelines: recommendations to the EC

To require brain health research promoters to conduct their R&I with a multi-stakeholder and co-accountable approach by engaging patients in their research agendas towards RRI
To provide adequate funding to support the patient engagement strategy in brain R&I projects
To encourage researchers working with patient organizations to enable the transition from individual to collective patients’ experiential knowledge
To recommend the use of metrics to evaluate the performance and effectiveness of patient engagement in research

### MULTI-ACT participatory and anticipatory governance: a new system for the assessment of research impact across different dimensions

One innovative feature of the MULTI-ACT CRIF versus existing models is that it considers the mission-related dimension as one explicit driver for accountability. The MULTI-ACT framework introduces the evaluation of the efficacy of an R&I initiative interpreted as its capacity to fulfil the shared mission (along with the other impact dimensions detailed hereafter) as a pivotal element to promote research programmes and projects that have an impact on patients and society. Around this core, the development of high-quality health research revolves (excellence), which has to be aligned with the mission success of health research (efficacy) and the co-participation of all the stakeholders who are directly or indirectly participating in the field (social), while enabling the economic and financial dimension (efficiency). The fifth dimension, patient-reported dimension, is transverse, to be applied across the other four dimensions. It considers investigating the impacts on patients and highlighting the active engagement of patients throughout the research process.

As an integral part of this framework, the MULTI-ACT MSC is a practical tool that fosters collective evaluation [57]. It can be used for collaborative decision-making towards a return on investment in research for the relevant stakeholders that best reflects the relevant claims and issues for the stakeholders relevant to the mission, including patients and society (e.g. informing the design and implementation of policies, development agendas or funding programmes). So far, most of the conventional metrics for measuring the impact of the research agendas on people's health have not been effective, lacking shared impact measures and support infrastructure to allow for true alignment of efforts and accountability of results. This has discouraged the true commitment of the various stakeholders and thus the impact of health research on healthcare. The MSC can be applied at the beginning or during the development of a research initiative to engage multiple stakeholders in collectively defining the impact indicators towards a given mission. MULTI-ACT has identified 53 aspects and 125 indicators that the MULTI-ACT digital toolbox makes available for multi-stakeholder research initiatives to develop an impact assessment scorecard [42, 58].

## Discussion

The MULTI-ACT project led to the development of a CRIF prototype. The institutional changes fostered by European RRI models such as MULTI-ACT aim to promote structural changes within research organizations and their ways of choosing, funding and performing research. The MULTI-ACT digital framework has a solid scientific foundation and represents a holistic management model encompassing mission-driven governance, patient engagement and impact assessment. The model is being used by several institutions [59] (i.e. Progressive Multiple Sclerosis Alliance, Multiple Sclerosis Care Unit, Global Patient Reported Outcomes for MS Initiative, Cluster for Epilepsy [EPI-CLUSTER] of the European Brain Research Area [EBRA], Horizon 2020 European project ALAMEDA: Bridging the Early Diagnosis and Treatment Gap of Brain Diseases via Smart, Connected, Proactive and Evidence-based Technological Interventions). These institutions now seek sustainability plans to exploit initial results and turn the MULTI-ACT prototype into an up-and-running management tool. Horizon Europe [60] can and should be seen as an opportunity to leverage the insights gained from the past decade of activities in RRI and to exploit them, particularly with regard to fair and equitable co-creation activities. In particular, in Horizon Europe, “Missions” are a key element and require inclusivity by enabling co-design and co-creation of and within “Missions”.

Globally, other relevant initiatives are shaping the field, catalysing a stronger shift towards a culture of participatory governance in research. Relevant initiatives are included and discussed in the MULTI-ACT landscape analysis [51]. Among these, MULTI-ACT CRIF can add value to the strategies of two other existing initiatives: the United States-based Patient-Centered Outcomes Research Institute (PCORI) and Canada's Strategy for Patient-Oriented Research (SPOR). The PCORI portfolio highlights in particular the need to develop validated measures to assess engagement processes and outputs from multiple perspectives. Patient-reported indicators included in the MULTI-ACT digital impact assessment system offer a unique opportunity to capture the experiential knowledge of patients and make it scientifically relevant for all stakeholders. The SPOR initiative has developed a patient engagement (PE) framework [61] that outlines key opportunities for “improving worthwhile collaborations in the identification of health research priorities as well as in the design and conduct of research projects”. Within the SPOR framework, the MULTI-ACT Engagement Coordination Team is an innovative governance body that ensures the representativeness of the patient community relevant to a given mission. Overall, we are working to integrate into the MULTI-ACT digital toolbox other relevant complementary tools belonging to other initiatives, in particular in respect to stakeholder mapping and scope [62].

The application of RRI managerial models, such as the MULTI-ACT CRIF, is particularly urgent in the field of brain health research. The investments made in research by the EC have been directed “at better understanding brain function and dysfunction, developing methods for diagnosis and monitoring, prevention, treatment as well as care and support” [63].

While advances in basic neuroscience research hold great promise, they also create the need for a unique brain research and care ecosystem capable of addressing societal needs related to brain conditions [64]. The development and employment of RRI participatory and anticipatory governance models and tools will be instrumental in meeting this challenge [65].

The application of the MULTI-ACT CRIF, made available to the R&I community through a free and user-friendly digital toolbox, will increase the impact of multi-stakeholder research initiatives on people with brain conditions through an innovative co-accountability strategy. The explicit drivers of the innovative co-accountability approach of MULTI-ACT are a mission-oriented approach that drives new guidelines to enable science with and of patient input, and a new system for the assessment of research impact across different dimensions.

In the brain conditions domain, engaging society and patients as key stakeholders, and measuring impact on outcomes that matter most to them, will give brain R&I the direction to make all the relevant stakeholders co-accountable for social needs and thus for the realization of groundbreaking fundamental research. This cannot be achieved through market innovation only [1]. We should prioritize a kind of innovation normatively underpinned by goals such as the Sustainable Development Goals of the United Nations Agenda 2030 [66]. And yet we have also seen, through for example the monumental efforts made to develop a vaccine for COVID-19, that innovation can be of critical importance to the health and future of our species. However, a recent Italian study, for example, indicates that “citizens and patients health engagement is positively related to the intention to vaccinate and that this relationship is partially mediated by the general attitude towards vaccines” [67]. We need to encompass inclusive and holistic approaches to solve challenging missions and make all stakeholders co-accountable and co-responsible for a given mission and shared agenda. This renewed research embedding RRI principles and based on other relevant models will continue to create societies and economies better prepared for crises such as COVID-19.
